# The Evolving Role of Immune Checkpoint Inhibitors in Hepatocellular Carcinoma Treatment

**DOI:** 10.3390/vaccines9050532

**Published:** 2021-05-20

**Authors:** Patrizia Leone, Antonio Giovanni Solimando, Rossella Fasano, Antonella Argentiero, Eleonora Malerba, Alessio Buonavoglia, Luigi Giovanni Lupo, Valli De Re, Nicola Silvestris, Vito Racanelli

**Affiliations:** 1 Unit of Internal Medicine “Guido Baccelli”, Department of Biomedical Sciences and Human Oncology, University of Bari Medical School, 70124 Bari, Italy; patrizia.leone@uniba.it (P.L.); antonio.solimando@uniba.it (A.G.S.); rossella.fasano.93@gmail.com (R.F.); ele94.malerba@gmail.com (E.M.); alessio.buonavoglia85@gmail.com (A.B.); n.silvestris@oncologico.bari.it (N.S.); 2 IRCCS Istituto Tumori “Giovanni Paolo II”, 70124 Bari, Italy; argentieroantonella@gmail.com; 3 Department of Experimental Diagnostic and Specialty Medicine, “L. and A. Seràgnoli”, University of Bologna, 40138 Bologna, Italy; 4 Department of General Surgery and Liver Transplantation, University of Bari, 70124 Bari, Italy; luigig.lupo@gmail.com; 5 Immunopathology and Cancer Biomarkers—Bio-Proteomics Facility, CRO Aviano National Cancer Institute, 33081 Aviano, Italy; vdere@cro.it

**Keywords:** hepatocellular carcinoma, immune checkpoint molecules, immune checkpoint inhibitors, immune microenvironment

## Abstract

Hepatocellular carcinoma (HCC) is one of most common cancers and the fourth leading cause of death worldwide. Commonly, HCC development occurs in a liver that is severely compromised by chronic injury or inflammation. Liver transplantation, hepatic resection, radiofrequency ablation (RFA), transcatheter arterial chemoembolization (TACE), and targeted therapies based on tyrosine protein kinase inhibitors are the most common treatments. The latter group have been used as the primary choice for a decade. However, tumor microenvironment in HCC is strongly immunosuppressive; thus, new treatment approaches for HCC remain necessary. The great expression of immune checkpoint molecules, such as programmed death-1 (PD-1), cytotoxic T-lymphocyte antigen 4 (CTLA-4), lymphocyte activating gene 3 protein (LAG-3), and mucin domain molecule 3 (TIM-3), on tumor and immune cells and the high levels of immunosuppressive cytokines induce T cell inhibition and represent one of the major mechanisms of HCC immune escape. Recently, immunotherapy based on the use of immune checkpoint inhibitors (ICIs), as single agents or in combination with kinase inhibitors, anti-angiogenic drugs, chemotherapeutic agents, and locoregional therapies, offers great promise in the treatment of HCC. This review summarizes the recent clinical studies, as well as ongoing and upcoming trials.

## 1. Introduction

Hepatocellular carcinoma (HCC) is one of the most common cancers and the fourth leading cause of cancer-related death worldwide [1]. It is more frequent in males between 30 and 50 years of age. Almost 85% of HCC cases occur in underdeveloped countries such as Eastern Asia and sub-Saharan Africa [2,3].

Commonly, HCC development occurs in a liver that is severely compromised by chronic injury or inflammation, associated with chronic infection with hepatitis B virus (HBV) or hepatitis C virus (HCV), the consumption of aflatoxins, frequent contaminants of foods and feeds that can act synergistically with HBV, and tobacco smocking [4,5,6]. Hepatocarcinogenesis is a multistep process starting with liver injury, followed by gene mutations, chronic local inflammation, fibrosis, cirrhosis, and cancer [7]. The introduction of anti-HBV vaccination and direct-acting viral (DAA) therapies for HCV treatment has led to the almost total eradication of the viruses, in terms of undetected viral nucleic acids. However, although HBV or HCV eradication is associated with the regression of fibrosis or cirrhosis, these patients might have a higher risk of developing HCC compared with pre-cirrhotic patients [8,9,10]. Moreover, the coexistence of hypertension, diabetes mellitus, obesity, and dyslipidemia further enhance HCC risk [11]. HCC can also develop in the context of a non-cirrhotic liver (NCL), due to alcohol abuse, drugs, autoimmune hepatitis, and non-alcoholic fatty liver disease (NAFLD) [12].

The tumor microenvironment in HCC is multifaceted and strongly influences tumor initiation and progression. Complex interactions between tumor cells and stromal cells, along with high levels of immunosuppressive cytokines, such as interleukin (IL)-10 and transforming growth factor (TGF)-β, promote the generation of an immunosuppressive milieu characterized by immune cell dysfunction and expansion of immunosuppressive cell populations such as regulatory T (Treg) cells and cells expressing checkpoint molecules, namely programmed cell death 1 (PD-1), programmed death ligand-1 (PD-L1), programmed death ligand-2 (PD-L2), cytotoxic T-lymphocyte antigen 4 (CTLA-4), lymphocyte activating gene 3 protein (LAG-3), and T cell immunoglobulin and mucin domain molecule 3 (Tim-3) [13]. Moreover, long-term exposure to antigens can cause an overexpression of the immunosuppressive checkpoint molecules on T cells, resulting in cell exhaustion, immune escape of tumor cells, and HCC development [13,14]. Unfortunately, HCC is a very aggressive tumor characterized by a rapid onset and progression [15]. The Barcelona Clinic Liver Cancer (BCLC) system is the current staging system for HCC that indicates the best candidate for the best therapy currently available, based on five HCC stages [16]. The very early stage, stage 0, includes patients with one asymptomatic nodule of less than 2 cm and well-preserved liver function. The early stage, stage A, corresponds to patients with a single tumor smaller than 3 cm. Patients with BCLC stages 0 and A are suitable for resection, transplantation, or ablation. The intermediate stage, stage B, encompasses patients with large or asymptomatic multinodular tumors confined to the liver parenchima. The advanced stage, stage C, comprises patients with symptomatic tumors and/or macrovascular invasion and extrahepatic spread. Patients with BCLC B and C are treated with transcatheter arterial chemoembolization (TACE), systemic therapy with sorafenib, or new agents in the setting of research trials. End stage, stage D, includes patients with a very poor prognosis who receive the best supportive care [4,17,18]. Additional treatment options for HCC are targeted therapies based on tyrosine protein kinase inhibitors [1,15,19] and histone deacetylase inhibitors (HDACIs) [20]. However, the prognosis remains dismal, with a five-year survival rate of ~12.5% [21].

Recent advances in tumor biology are currently attracting great interest in immune checkpoint inhibitors (ICIs) for the treatment of advanced HCC. Combination therapies using ICIs with other agents are estimated to overcome the immunosuppressive tumor microenvironment and to improve the prognosis of HCC patients.

## 2. Liver, Inflammation and Cancer

**Figure 1:**
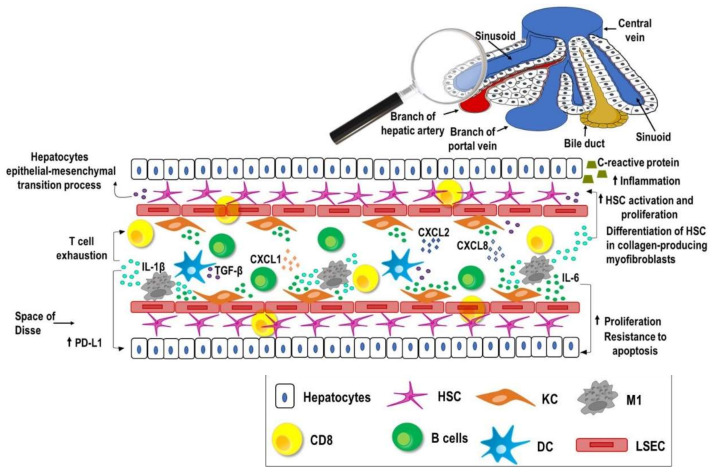
The liver as an immunological organ. Because of its distinctive structure and specific blood supply route, the liver preserves a unique immune microenvironment. The liver sinusoidal endothelial cells (LSECs) form the fenestrated wall of the liver sinusoid and control the trafficking of molecules and cells from the liver parenchyma to the blood. At the sinusoids, circulating lymphocytes interact with antigens presented by endothelial cells, Kupffer cells (KCs), and liver resident dendritic cells (DCs), and, through fenestrations, they can access to the Disse space to get in touch with hepatocytes and hepatic stellate cells (HSCs). During liver injury or infection, a local inflammatory response is triggered as a result of the release of inflammatory cytokines (IL-6, IL-1β, and TGF-β) and chemokines (CXCL1, CXCL2, and CXCL8), leading to tissue damage and HCC development. Specifically, KCs and M1 macrophages produce IL-6 and TGF-β, which induce hepatocytes proliferation and resistance to apoptosis, and trigger the hepatocytes epithelial–mesenchymal transition (EMT) process and the differentiation of HSCs in collagen-producing myofibroblasts that promote fibrosis. Hepatocytes are mainly responsible for the production of the C-reactive protein supporting inflammation. M1 macrophages also release IL-1β, which enhances PD-L1 expression on hepatocytes resulting in T cells exhaustion and tumor development.

The liver can be defined as an immunological organ because of its unique structure and organization, which promotes the direct or indirect priming of lymphocytes, modulates immune response, and maintains a local and systemic immune tolerance to self and foreign antigens [5,22]. The blood, rich in nutrients and antigens, coming from the gastrointestinal tract, enters the liver via the portal vein, crosses the hepatic sinusoids, and leaves the liver via the central hepatic veins [5,23]. At the sinusoids, the blood is scanned by antigen presenting cells and lymphocytes (Figure 1); indeed, because of the small sinusoid diameter, circulating lymphocytes can make contact with antigens presented by endothelial cells, Kupffer cells (KCs), and liver resident dendritic cells (DCs). Moreover, taking advantage of the fenestration of the liver sinusoidal endothelial cells (LSECs), circulating lymphocytes can gain access to the Disse space and interact with hepatocytes and hepatic stellate cells (HSCs).

Under physiological conditions, the liver is continuously exposed to gut derived microbial products, which are tolerated by the hepatic immune system, while, at the same time, it is ready to fight against possible infectious agents, tumor cells, or tissue damage. Homeostatic, tightly controlled inflammation is established, which is linked to mechanisms that promote tissue regeneration [24]. Failure to clear “dangerous” stimuli leads to pathological inflammation, dysregulation of the immune system, and disruption of tissue homeostasis characterized by a progressive development of fibrosis, cirrhosis, and cancer [7]. HCC is a good example of inflammation-related cancer; in fact, 90% of HCC cases are associated with inflammation [25]. During liver injury or infection, a powerful protective response is triggered by a network of cytokines, chemokines, and growth factors involved in various interconnected inflammatory signaling pathways, whose dysregulation leads to liver cancer [26,27].

IL-6, IL-1β, and TGF-β are the main cytokines involved in inflammation and HCC development [28,29]. The serum levels of IL-6 are higher in HCC patients than in healthy donors [30]. IL-6 is mostly released by KCs, supports a local inflammatory response, and promotes hepatocytes proliferation and resistance to apoptosis via JACK/STAT3 pathway activation [31].

IL-1β, which stimulates the production of the C-reactive protein implicated in the inflammatory process [32], enhances the activation and proliferation of HSCs and their differentiation in collagen-producing myofibroblasts that promote fibrosis [33]. Moreover, IL-1β released by M1 macrophages induces PD-L1 expression on HCC cells, which interact with PD-1 on T cells, thus promoting T cells exhaustion and tumor progression [34].

TGF-β induces the hepatocyte destruction and activation (transdifferentiation) of hepatic stellate cells and fibroblasts to myofibroblasts with subsequent extracellular matrix deposition and fibrogenesis. TGF-β also triggers an epithelial–mesenchymal transition (EMT) process in hepatocytes that may participate, directly or indirectly, in myofibroblasts increase [35]. TGF-β also enhances the expression of the epidermal growth factor receptor (EGFR) and its ligands, like EGF and TGF-α, involved in the regenerative and protective natural response of the liver to acute tissue injury, and, when deregulated, contribute to neoplastic transformation. Indeed, their upregulation is associated with aggressive phenotypes and poor survival in HCC patients [26,36].

Sustained hepatic inflammation as a result of several types of injury (e.g., hepatitis C or nonalcoholic steatohepatitis) also activates distinct chemokine pathways that regulate the migration and functions of hepatocytes, Kupffer cells, hepatic stellate cells, endothelial cells, and circulating immune cells. The damage to hepatocytes results in the release of pathogen- or danger-associated molecular patterns, which interact with the Toll-like receptors (TLRs), in particular TLR4 expressed on the KCs and HSCs, stimulating the secretion of cytokines, such as TNF-α, IL-6, IL-1β, and TGF-β, and chemokines, such as CXCL1, CXCL2, and CXCL8. This results in migration of macrophages and neutrophils in the liver, with consequent amplification of the inflammatory response and tumor development [37].

Moreover, the liver is a strong immunosuppressive microenvironment characterized by high levels of immunosuppressive cytokines, such as IL-10 and TGF-β, released by LSECs and Treg cells, and has a great expression of immune checkpoint molecules, PD-1, CTLA-4, lymphocyte activating gene 3 protein (LAG-3), and mucin domain molecule 3 (TIM-3) on immune cells [38,39,40,41,42].

## 3. Immune Checkpoints

Several studies have highlighted the important role of immune checkpoints PD-1, CTLA-4, LAG-3, and TIM-3 in HCC development [38,39,40,41,42].

**Figure 2:**
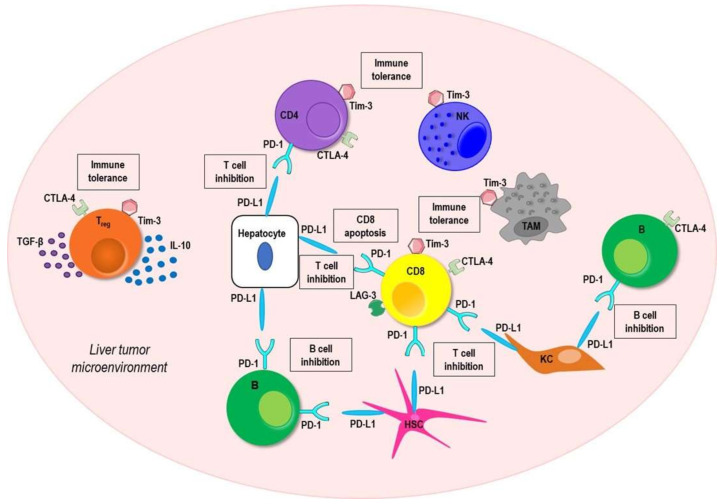
Schematic illustration of immune inhibitory interactions in the liver hepatocellular carcinoma microenvironment. Tumor-specific infiltrating CD4 T helper cells exhibit a great expression of immune checkpoint molecules programmed cell death-1 (PD-1), cytotoxic T-lymphocyte antigen 4 (CTLA-4), and mucin domain molecule 3 (TIM-3), whereas tumor-specific infiltrating CD8 T cells show a high expression of PD-1, CTLA-4, TIM-3, and lymphocyte activating gene 3 protein (LAG-3) on their surface. High levels of PD-1 and CTLA-4 are also displayed on the B cell surface. The interaction between PD-1 and its ligand PD-L1 expressed on hepatocytes, Kupffer cells (KCs), and hepatic stellate cells (HSCs) promotes T and B cell inhibition and induces CD8 T cells apoptosis. CTLA-4 inhibits immune functions favoring tumor growth. TIM-3 is involved in immune response regulation and immune tolerance induction. Besides T cells, tumor associated macrophages (TAMs) and natural killer (NK) cells markedly express TIM-3.

PD-1, CTLA4, LAG-3, and TIM-3 were upregulated on CD4^+^ and CD8^+^ T cells in HCC tissue and peripheral blood [39,41,43,44,45] (Figure 2). Interestingly, tumor-specific infiltrating T helper cells showed a drastic upregulation of PD-1, TIM-3, and CTLA4 expression; whereas tumor-specific infiltrating cytotoxic T cells exhibited a significant increase of PD-1, TIM-3, LAG-3, and CTLA4 expression, compared with their counterparts in tumor-free liver tissues and in blood [46]. Moreover, the interaction between PD-1 on tumor infiltrating cytotoxic CD8^+^ T cells and its ligand PD-L1 on hepatocytes induces CD8^+^ T cells apoptosis. PD-1 upregulation on circulating and intratumor CD8^+^ T cells could also predict a poorer disease progression and postoperative recurrence [41].

PD-1 is highly expressed by a protumorigenic B cell subset in HCC patients. These PD-1^+^ B cells displayed a unique phenotype (CD5^(hi)^CD24^(−/+)^CD27^(hi/+)^CD38^(dim)^), different from the conventional phenotype of the peripheral regulatory B cells (CD24^(hi)^CD38^(hi)^) [42].

PD-L1 is overexpressed on hepatocytes, KCs, and HSCs cells, and interacting with PD-1 on T and B cells determines T and B cell inhibition. Furthermore, stimulation with type I or type II interferons strongly enhanced PD-L1 expression, which was correlated with tumor aggressiveness, vascular invasion, and poor survival [47,48,49].

CTLA-4 is strongly expressed on Treg cells, which are very abundant, fully differentiated, and highly activated in the livers of HCC patients [50,51].

TIM-3 is a far less examined immune checkpoint molecule that is involved in immune response regulation and immune tolerance induction [52]. Besides T cells, Treg, tumor associated macrophages, and NK cells markedly expressed TIM-3, and their expression was correlated with a poor prognosis in HCC patients [40].

Interestingly, Tan et al. demonstrated Tim-3 upregulation on conventional and liver resident NK cells that hampered their cytokine secretion and cytotoxicity in a PI3K/Akt/mTOR-dependent manner and the blockade of Tim-3 boosted the antitumor immunity [53].

## 4. Immune Checkpoint Inhibitors

Multi-tyrosine kinases inhibitors (TKIs), such as sorafenib, have been used as a primary choice for a decade. However, improvements in the overall survival rate have been unsatisfactory. Moreover, unlike other targeted therapies, predictive and prognostic markers in HCC subjects treated with sorafenib are scanty [54]. In addition, the tumor microenvironment in HCC is strongly immunosuppressive; thus, new treatment approaches for HCC remain necessary. The introduction of ICIs, used as single agents (Table 1) or in combination therapy (Table 2), to regulate the balance of immune homeostasis represents a clinical breakthrough [55,56].

### 4.1. Monotherapy

Nivolumab is a PD-1-blocking fully human IgG4 monoclonal antibody (mAb) that enhances the activity of effector T cells, enabling them to recognize and attack cancer cells in an HCC microenvironment. The food and drug administration (FDA) approved the use of nivolumab for advanced HCC patients in September 2017 based on the results of the CheckMate 040 clinical trial (NCT01658878). This study was a multicenter, phase I/II, open-label, non-comparative, dose escalation, and expansion trial conducted in patients with advanced HCC, with or without chronic viral hepatitis (HCV or HBV), who were previously treated with sorafenib or left untreated. During the dose escalation phase, patients received a drug dose of 0.1–10 mg/kg once every 2 weeks. During the dose expansion phase, patients were treated with a drug dose of 3 mg/kg once every 2 weeks.

In both phases, nivolumab exhibited a manageable safety profile with adequate tolerability and an incidence of treatment-related adverse events that was not associated with the dose. Moreover, the dose expansion phase showed durable objective responses with objective response rates of 15–20%, a median overall survival of 15.6 months, and a significant tumor reduction [57].

Pembrolizumab is another anti-PD-1 mAb that has been evaluated in the KENYOTE-224 trial (NCT02702414), a phase II, single-arm, non-randomized study. In this study, 104 patients were enrolled with advanced HCC, previously treated with sorafenib (cohort 1) or with no history of systemic treatment (cohort 2). Patients received intravenous pembrolizumab (200 mg) at 3-week intervals for 2 years or until disease progression. The objective response rate was 17%, with one patient (1%) reaching complete response, 17 patients (16%) with partial responses, 46 patients (44%) had stable disease, and 34 patients (33%) showed progression. Treatment-related adverse events developed in 76 (73%) of the 104 patients, of which 16 were severe (15%) [58]. Pembrolizumab was further assessed as a second-line treatment in HCC patients in the KENYOTE-220 study, a randomized, double-blind, placebo-controlled, phase III trial that confirmed the results obtained with the KEYNOTE-224 study [84].

Camrelizumab is an anti-PD-1 mAb with a different binding epitope to nivolumab and pembrolizumab. A multicenter, open-label, parallel-group, randomized, phase II trial (NCT02989922) aimed to examine the antitumor activity and safety of camrelizumab in advanced Chinese HCC patients who progressed on or were intolerant to previous systemic treatment. The enrolment of the study is closed, but the follow-up is ongoing. The results presented a manageable drug toxicity; an objective response of 14.7%; and a 6-month and 12-month overall survival probability of 74.4% and 55.9%, respectively, suggesting the potential use of camrelizumab as a new second-line therapeutic option for patients with advanced HCC [59].

Tislelizumab and sintilimab are anti-PD1 mAbs undergoing a phase III trial [55]. The Rationale-301 (NCT03412773) is a randomized, open-label, multicenter study that will evaluate the efficacy and safety of tislelizumab vs. sorafenib as a first-line treatment in patients with advanced unresectable HCC [60]. The combination of sintilimab with the anti-VEGF Ab bevacizumab is being evaluated in the ORIENT-32 study (NCT03794440), which will be described in the next section.

Durvalumab, atezolizumab, and avelumab are anti-PD-L1 mAbs undergoing development [55]. Evaluation of durvalumab monotherapy for HCC patients in a phase I/II trial (NCT01693562) showed an acceptable safety profile and promising antitumor activity [61]. The combination of durvalumab (anti-PD-L1 mAb) and tremelimumab (an anti-CTLA-4 mAb) confirmed its safety and will be discussed in the next section [65,85].

Tremelimumab is the first anti-CTLA-4 fully human IgG2 mAb used in HCC treatment. A phase II, non-controlled, open-label, multicenter clinical trial (NCT01008358) revealed a safety profile, as well as antitumor and antiviral effects, associated with an enhanced specific anti-HCV immune response in patients with HCC and HCV infection [62].

Cabolimab (TSR-022) is an anti-Tim-3 mAb, whose efficacy as a single agent and in combination with anti-PD1, anti LAG-3, or anti PD-L1 mAbs is under investigation in a multicenter, open-label, first-in-human study (NCT02817633) in patients with advanced solid tumors, including HCC [63]. In the last years, other molecules of anti-Tim-3, like MBG453 (mAb) and Symo23 (Recombinant human Ab), have been developed and several trials are ongoing to evaluate their effectiveness [86,87].

INCAGN02385 is an anti-LAG-3 mAb that has generated great interest. Evaluation in cynomolgus monkeys described enhanced T cell responsiveness to TCR stimulation alone or in combination with PD-1/PD-L1 axis blockade with a good safety profile [88]. These results supported the assessment of INCAGN02385 in patients with advanced or metastatic solid tumors. A phase I, open-label, dose-escalation trial (NCT03538028) investigated the safety, tolerability, and preliminary efficacy of INCAGN02385 in patients with advanced malignancies, including HCC, but these results have not yet been published [64].

**Figure 3:**
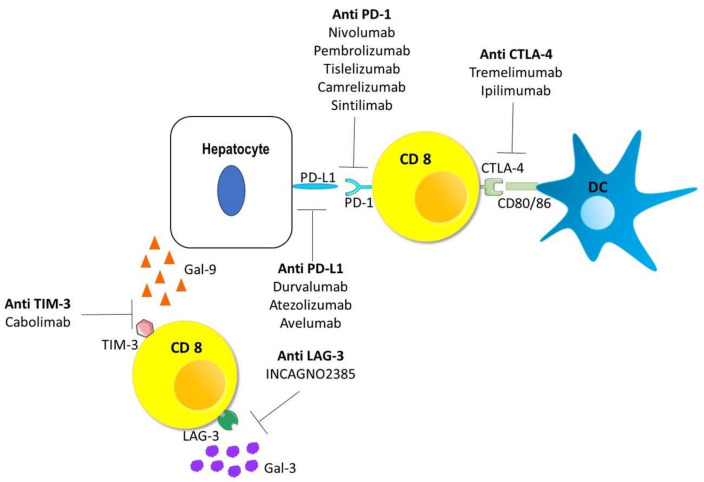
Schematic diagram of immune checkpoint expressions and their inhibitors.

A summary about ICIs and their blocking capacities is provided in Figure 3.

### 4.2. Combination Therapy

**Figure 4:**
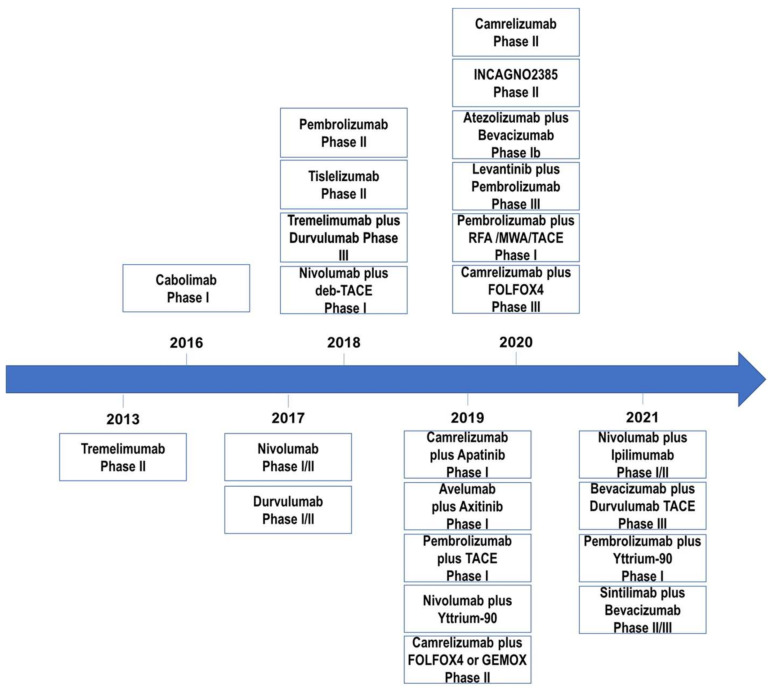
Development and clinical trials of immune checkpoint inhibitors as single agents or in combination with other treatments for HCC from 2013 to 2021.

To date, ICI monotherapy has shown potential therapeutic effects in HCC, but so far, randomized phase III trials have not revealed their superiority in terms of survival over standard treatments [84,89]. Systematic treatment for advanced HCC has changed drastically in the last years (Figure 4). Immuno-oncology combination therapies for advanced HCC achieved promising results, with low cytotoxicity and durable responses. Attention has been focused on combinations of two ICIs, combination of ICIs with inhibitors of angiogenesis, locoregional therapies, or chemotherapies (Table 2).

#### 4.2.1. Combinations of Two Immune Checkpoint Inhibitors

The CheckMate 040 randomized clinical trial, a multicenter, open-label, multicohort study (NCT01658878), demonstrated the efficacy of the combination of the anti-PD-1 mAb nivolumab with the anti-CTLA-4 mAb ipilimumab in phase I/II. Compared with nivolumab monotherapy, the combination nivolumab plus ipilimumab ameliorated clinical outcomes with a higher objective response rate and a durable response in patients with advanced HCC previously treated with sorafenib. Moreover, the combined treatment displayed a manageable toxicity, with no new safety signals. Based on these results, in March 2020, the FDA approved the combination therapy of the arm A regimen (four doses nivolumab 1 mg/kg plus ipilimumab 3 mg/kg every 3 weeks, then 240 mg nivolumab every 2 weeks) as a second-line treatment after sorafenib [66].

Two other clinical studies of nivolumab (anti-PD-1 mAb) plus ipilimumab (anti-CTLA-4 mAb) as a neoadjuvant therapy are ongoing. The first is a phase II trial (NCT03222076) comparing nivolumab as a monotherapy with nivolumab plus ipilimumab treatment in patients with liver cancer that can be removed by surgery [67]. The aims of this trial are to evaluate the safety and tolerability of this treatment and to assess the efficacy of presurgical nivolumab alone or nivolumab plus ipilimumab therapy in HCC patients by estimating the objective response rates and time to progression. The second is a phase II trial (NCT03510871) in Taiwan, and is examining only the combination therapy [68].

The HIMALAYA study (NCT03298451), an ongoing open-label, multicenter, randomized phase III trial, is evaluating the efficacy of tremelimumab (anti-CTLA-4 mAb) plus durvalumab (anti-PD-L1 mAb) in the treatment of patients with no prior systemic therapy for unresectable HCC. The study showed promising results regarding the survival and anti-tumor response [65].

#### 4.2.2. Combination of Immune Checkpoint Inhibitors with Inhibitors of Angiogenesis

An open-label, multicenter, multiarm, phase 1b study (GO30140, NCT02715531) assessed the efficacy and safety of the combination of atezolizumab (anti-PD-L1 mAb) and bevacizumab (anti-VEGF mAb) in patients with unresectable HCC who had not received any previous systemic therapy [69]. The study showed a significant improvement of progression free survival (PFS) and a reduction in the risk of progression or death with atezolizumab plus bevacizumab compared with atezolizumab monotherapy [90]. This combination therapy has been compared with standard of care sorafenib in a global, multicenter, open-label, phase III randomized trial (IMbrave150, NCT03434379). This study revealed a significantly better overall survival and PFS outcomes with atezolizumab plus bevacizumab than with sorafenib (the estimated rates of survival at 6 months was 84.8% vs. 72.2% and median PFS, 6.8 months vs. 4.3 months). Moreover, the response rate was 27.3% (95% CI, 22.5 to 32.5) with atezolizumab plus bevacizumab and 11.9% (95% CI, 7.4 to 18.0) with sorafenib, according to an independent assessment using the Response Evaluation Criteria in Solid Tumors, version 1.1 (RECIST 1.1) [70].

Based on the results of the REFLECT study, which approved lenvatinib (anti-VEGFR mAb) as a first-line treatment for advanced HCC patients [91], an open-label multicenter single-arm phase Ib study (NCT03006926) evaluated the efficacy of the combination of lenvatinib (anti-VEGFR mAb) with pembrolizumab (anti-PD-1 mAb) in unresectable HCC. Patients showed a favorable toxicity profile and high response rates due to an improvement in the antitumor activity [71]. The FDA has defined the treatment “revolutionary” for the first-line therapy of patients with unresectable HCC and not susceptible to locoregional therapy. Therefore, an ongoing double-blind randomized controlled phase III study (NCT03713593) is comparing the lenvatinib plus pembrolizumab combination with the lenvatinib plus placebo combination, and should confirm the efficacy and safety of this combined therapy [72].

An alternative combination is camrelizumab (anti-PD-1 mAb) plus apatinib (anti-VEGFR2 mAb). An open-label, dose-escalation (phase Ia) and expansion study (phase Ib) (NCT02942329) revealed encouraging results, with 50% of patients with HCC achieving a partial response and manageable toxicity [73].

The ORIENT-32 study (NCT03794440) is a randomized, open-label, multicenter trial in China that will compare sintilimab (anti-PD1 mAb) and bevacizumab (anti-VEGF mAb) vs. sorafenib as a first-line treatment in patients with advanced HCC [74].

A phase I ongoing trial (NCT03289533) is evaluating the safety and tolerability of avelumab (anti-PD-L1 mAb) in combination with axitinib (anti-VEGF mAb) as a first line treatment in patients with advanced HCC [75].

#### 4.2.3. Combination of Immune Checkpoint Inhibitors with Locoregional Therapies

Given that the conventional locoregional therapies induce the release of local inflammatory factors and neoantigens, several trials are assessing ICIs as an adjuvant therapy in combination with TACE and radiofrequency ablation (RFA). The preliminary results have shown that this combination strategy promotes anti-tumor T cell response and reduction of Treg [92].

One prospective, non-randomized study demonstrated that RFA, TACE, and chemoablation (CA) stimulated a CD8 T cell response and increased the efficacy of the ICI tremelimumab (anti-CTLA-4 mAb) in patients with advanced-stage HCC. Moreover, this combination showed no dose-limiting toxicity and an intriguing clinical activity with a partial response rate of 26% and overall survival of 12.3 months [93].

At present, several ongoing studies are evaluating the combination of TACE with ICIs. A multicenter, non-randomized, pilot study (NCT03143270) is testing the safety and feasibility of the drug eluting bead transarterial chemoembolization (deb-TACE) in combination with nivolumab (anti-PD-1 mAb) in subjects with advanced HCC [76]. Another ongoing trial, the EMERALD-1 study (NCT03778957), has recently been proposed to evaluate the combination of bevacizumab (anti-VEGF mAb) and durvulumab (anti-PD-L1 mAb) with TACE in patients with HCC [77].

The preliminary results of an open label, single arm, multicenter study (NCT03397654) encouraged the clinical development of the combination of pembrolizumab (anti-PD-1 mAb) and TACE in patients with intermediate-stage HCC. This study is revealing a tolerable safety profile with no evidence of synergistic toxicity [78]. Pembrolizumab is also currently under evaluation as an adjuvant therapy after RFA, microwave ablation (MWA), or brachytherapy, or a combination of TACE with RFA, MWA, or brachytherapy in HCC patients. This is a multicenter, single arm, prospective, open-label phase II trial (IMMULAB study, NCT03753659) that will be completed in 2023. Early clinical data indicate an acceptable safety profile [79]. Furthermore, an open-label, multicenter, ongoing trial (NCT03099564) is assessing the safety and the effects of pembrolizumab (anti-PD-1 mAb) plus the radiology procedure with yttrium-90 in patients with a poor prognosis of HCC, not eligible for liver transplant or surgical resection [80]. The use of yttrium-90 is also being evaluated in combination with nivolumab (anti-PD-1 mAb) for the treatment of HCC patients undergoing surgical resection (NCT03812562) [81].

#### 4.2.4. Combination of Immune Checkpoint Inhibitors with Chemotherapies

The combination of immunotherapies with the conventional chemotherapeutic approaches represents a goal for HCC treatment. A multicenter, phase III, randomized, double-blinder study (NCT03605706) is evaluating the combination of camrelizumab (anti-PD-1 mAb) with FOLFOX4 regimen (fluorouracil+calcium folinate+oxaliplatin) compared with placebo plus FOLFOX4 in patients with advanced HCC who have never received prior systemic treatment. The results have not yet been reported, because the study is estimated to be completed in December 2021 [82]. Moreover, an open-label, non-randomized, phase II study (NCT03092895) is assessing the combination of camrelizumab with the FOLFOX4 regimen or GEMOX (gemcitabine and oxaliplatin) as first-line treatment in patients with advanced primary liver cancer or biliary tract carcinoma. The early results show that this combination is tolerable and might offer a new promising choice for the treatment of advanced HCC [83].

## 5. Side Effects

ICI therapy can cause an unbalance of the immune system, resulting in a wide range of immune-related adverse events (irAEs) that may affect a varied array of organ systems. These effects are commonly mild and manageable, but are sometimes life-threatening. Moreover, when HCC develops in the context of liver cirrhosis due to viral hepatitis and non-alcoholic steatohepatitis, these underlying diseases can overlap with irAEs and increase their severity, leading to consequences such as late recognition, inadequate work-up, and inappropriate treatment [94]. However, clinical experience with these agents has shown that the prompt recognition and appropriate management of irAEs can reduce serious complications.

The skin is the most common site of irAEs among patients receiving ICI therapy. Rash and pruritus are the most common clinical features, which generally appear within 2 weeks of treatment onset. Other common adverse reactions are diarrhea and colitis, hepatotoxicity with elevated AST, elevated alkaline phosphatase, and elevated ALT, and thyroid dysfunction usually manifests as hyperthyroidism and hypothyroidism [95].

Among HCC patients treated with the PD-1 inhibitors nivolumab and pembrolizumab as single agents, the incidence of rash, pruritus, and diarrhea was 10–23%, 12–19%, and 10–11%, respectively [95]. A special irAE, namely, reactive cutaneous capillary endothelial proliferation (RCCEP), is related to camrelizumab treatment [96]. Hypothyroidism occurs in 5–6% of patients during pembrolizumab treatment and 9% of patients during camrelizumab treatment [95]. Among the patients who received nivolumab and ipilimumab combination therapy, the incidence of pruritus, rash, and diarrhea was of 30–45%, 17–29%, and 12–24%, respectively [95]. Hypothyroidism occurs in 8–20% of patients and hyperthyroidism in 6–10% of patients [95]. In the phase Ib clinical trial of combination treatment with atezolizumab–bevacizumab, the incidence of rash and pruritus was 20% and 5%, respectively [69]. This combination therapy is also associated with hypertension, a known adverse effect of bevacizumab; diarrhea; and thyroid dysfunction [95]. Colitis was described in 10–12% of patients receiving anti-CTLA-4 treatment, 1% of patients receiving anti-PD-L1 treatment, and 14% of patients receiving combination therapies. Colitis arising may be related to the basic composition of the gut microbiota; severe cases can result in fatal colonic perforation and peritonitis [95]. Liver-related adverse events occur 4–12 weeks after beginning treatment. Cases of acute hepatic failure have been described after the administration of CTLA-4 and PD-1 inhibitors in both monotherapy and combination strategies [95].

Cardiac, haematological, pulmonary, neurological, renal, and ocular complications are rare ICI-related adverse events in patients with HCC [97].

Cardiac toxicity clinically manifests as dyspnoea, palpitations, or symptoms of congestive heart failure. Among the cardiac pathologies associated with ICIs, myocarditis is one of the most common. Haematological complications are often asymptomatic and include neutropenia, autoimmune haemolytic anaemia, immune thrombocytopenia, and aplastic anaemia. Pneumonia is a relatively rare irAE that is potentially life-threatening and for which early diagnosis and classification are essential in order to improve prognosis. Peripheral neuropathies, encephalopathy, myasthenia gravis, myelitis, meningitis, and Guillain–Barré syndrome are the principal complications related to the nervous system. Acute renal failure can include granulomatous tubulointerstitial nephritis and thrombotic microangiopathy. Ocular irAEs encompass inflammation of the eye, which can include uveitis, episcleritis, conjunctivitis, and orbital myopathy [97].

## 6. Future Direction

The goal of future therapy for HCC will be a combination of personalized treatments to target multiple pathways in the HCC cascade and to achieve tailored therapies improving the overall survival.

In the last few years, several preclinical studies have combined the use of chimeric antigen receptor T (CAR-T) cells with ICIs to overcome the highly immunosuppressive microenvironment, achieving promising results. Guo et al. produced PD-1-deficient CAR-T cells using a CRISPR/Cas9 system and demonstrated that the disruption of PD-1 increased the CAR T cell ability to kill HCC cells in vitro and in vivo [98].

Recent studies of HCC tumor xenografts in mice and in vitro established that engineered CAR-T cells targeting the glypican 3 (GPC3) molecule, highly expressed in HCC samples and absent in healthy liver samples, could attack and eliminate GPC3-positive HCC cells [99,100,101,102]. Phase I clinical studies aimed at evaluating the safety and efficacy of CAR-GPC3 T-cell therapy alone (NCT03980288, NCT04121273, and NCT03884751) or in combination with cyclophosphamide and fludarabine (NCT02905188), or cytokine-based treatment options (NCT04093648 and NCT03198546) are currently ongoing [103].

The next few years will be promising for drug development in HCC, both in terms of novel therapeutics and in terms of new combination strategies. For instance, the combination of a cancer vaccine and an ICI could also be an interesting strategy for the treatment of HCC patients that do not respond to single-agent ICI treatment because of the lack of tumor-infiltrating effector T cells. These different treatments might function synergistically; the vaccine might increase the number of tumor-infiltrating effector T cells and the anti-PD-1 might make these cells effective, leading to an active antitumor immune response [104,105]. Clinical trials to test such combinations are warranted.

## Figures and Tables

**Table 1:** Clinical trials investigating immune checkpoint inhibitor monotherapy in hepatocellular carcinoma.

Trial ID	Treatment	Target	Phase	Patient Number	Lines of Therapy	Status	Ref.
NCT 01658878	Nivolumab	PD-1	I/II	262	First/Second-line	Completed	[57]
NCT 02702414	Pembrolizumab	PD-1	II	104	Second-line	Completed	[58]
NCT 02989922	Camrelizumab	PD-1	II	220	Second-line	Completed	[59]
NCT 03412773	Tislelizumab	PD-1	III	674	First-line	Recruiting	[60]
NCT 01693562	Durvulumab	PD-L1	I/II	1022	First-line	Completed	[61]
NCT 01008358	Tremelimumab	CTLA-4	II	21	First-line	Completed	[62]
NCT 02817633 (part 1a)	Cabolimab	Tim-3	I	369	NA	Recruiting	[63]
NCT 03538028	INCAGN02385	Lag-3	I	22	NA	Completed	[64]

PD-1—programmed cell death 1; PD-L1—programmed death ligand-1; CTLA-4—cytotoxic T-lymphocyte antigen 4; Tim-3—T cell immunoglobulin and mucin domain-containing protein 3; Lag-3—lymphocyte-activation gene 3.

**Table 2:** Clinical trials investigating immune checkpoint inhibitor combination therapy in hepatocellular carcinoma.

Trial ID	Treatment	Target	Phase	Patient Number	Lines of Therapy	Status	Ref.
**ICI + ICI**							
NCT 03298451	Tremelimumab + Durvalumab	CTLA-4 PD-L1	III	1504	First- line	Recruiting	[65]
NCT 01658878	Nivolumab + Ipilimumab	PD-1 CTLA-4	I/II	148	Second-line	Recruiting	[66]
NCT 03222076	Nivolumab + Ipilimumab	PD-1 CTLA-4	II	30	NA	Recruiting	[67]
NCT 03510871	Nivolumab + Ipilimumab	PD-1 CTLA-4	II	40	NA	Recruiting	[68]
**ICI + angiogenesis inhibitor**							
NCT 02715531	Atezolizumab + Bevacizumab	PD-L1 VEGF	Ib	23	Second-line	Recruiting	[69]
NCT 03434379	Atezolizumab + Bevacizumab	PD-L1 VEGF	III	480	First- line	Recruiting	[70]
NCT 03006926	Lenvatinib + Pembrolizumab	VEGFR PD-1	Ib	104	First- line	Recruiting	[71]
NCT 03713593	Levantinib + pembrolizumab	VEGFR PD-1	III	750	First- line	Recruiting	[72]
NCT 02942329	Camrelizumab + Apatinib	PD-1 VEGFR2	I	14	Second-line	Recruiting	[73]
NCT 03794440	Sintilimab + Bevacizumab	PD-1 VEGF	II/III	595	First- line	Recruiting	[74]
NCT 03289533	Avelumab+ Axitinib	PD-L1 VEGF	I	22	First- line	Recruiting	[75]
**ICI + locoregional therapy**							
NCT 03143270	Nivolumab + deb-TACE	PD-1	I	14	NA	Recruiting	[76]
NCT 03778957	Bevacizumab + Durvalumab + TACE	VEGF PD-L1	III	710	NA	Recruiting	[77]
NCT 03397654	Pembrolizumab + TACE	PD-1	Ib	26	NA	Recruiting	[78]
NCT 03753659	Pembrolizumab + RFA or MWA or TACE	PD-1	II	30	NA	Recruiting	[79]
NCT 03099564	Pembrolizumab + Yittrium-90	PD-1	NA	30	NA	Recruiting	[80]
NCT 03812562	Nivolumab + Yittrium-90	PD-1	I	2	NA	Recruiting	[81]
**ICI + chemotherapy**							
NCT 03605706	Camrelizumab + fluorouracil+ calcium/orfolinate+ oxoliplatin	PD-1	III	396	First-line	Recruiting	[82]
NCT 03092895	Camrelizumab + Apatinib or fluorouracil+ calcium/folinate+ oxoliplatin or gemcitabine + oxoliplatin	PD-1	II	152	NA	Recruiting	[83]

PD-1—programmed cell death 1; PD-L1—programmed death ligand-1; CTLA-4—cytotoxic T-lymphocyte antigen 4; Tim-3—T cell immunoglobulin and mucin domain-containing protein 3; Lag-3—lymphocyte-activation gene 3; VEGF—vascular endothelial growth factor; VEGFR—vascular endothelial growth factor receptor; TACE—transarterial chemoembolization; deb-TACE—drug eluting bead transarterial chemoembolization; RFA—radiofrequency ablation; MWA—microwave ablation.
